# HIV infection, and overweight and hypertension: a cross-sectional study of HIV-infected adults in Western Kenya

**DOI:** 10.1186/s41182-020-00215-w

**Published:** 2020-05-07

**Authors:** Akiko Saito, Mohamed Karama, Yasuhiko Kamiya

**Affiliations:** 1 School of Tropical Medicine & Global Health, Nagasaki University, 1-12-4, Sakamoto, Nagasaki, 852-8523 Japan; 2 Center for Public Health Research, Kenya Medical Research Institute, Nairobi, Kenya

**Keywords:** HIV, AIDS, Overweight, Obesity, Abdominal obesity, Hypertension, Body image, Kenya

## Abstract

**Background:**

Non-communicable diseases (NCDs) are increasing in Kenya, where HIV/AIDS remains a leading cause of death; however, few studies have investigated obesity and hypertension among adults with HIV infection. We conducted a cross-sectional study in Homa Bay, Western Kenya, during 2015 to determine the prevalence of overweight/obesity and hypertension among HIV-infected adults and to identify their risk factors.

**Results:**

Anthropometric measurements and a structured questionnaire were administered to adults with HIV infection receiving care at Mbita Sub-county Hospital. A total of 251 HIV-positive individuals were enrolled. More women were overweight (17.2%) and obese (3.6%) than underweight (8.3%). The prevalence of abdominal obesity was high in women (62.7%), especially those aged 30–39 years. The prevalence of hypertension was 9.8% and 11.8% in men and women, respectively. Male participants tended to develop hypertension at an early age. Multivariate analysis showed that female sex was significantly associated with abdominal obesity. Regarding clinical factors, we identified an association between overweight and a history of opportunistic infections, as well as between hypertension and World Health Organization clinical stage. Sixty percent of HIV-infected participants assumed that a very thin body size indicated HIV infection.

**Conclusions:**

The main findings of this study include a greater prevalence of overweight than underweight as well as a high prevalence of abdominal obesity among women. Social perception toward body size among people with HIV infection might remain problematic. Individuals living with HIV in Kenya should receive preventive intervention for overweight and abdominal obesity, with consideration of relevant social and cultural aspects.

## Introduction

Non-communicable diseases (NCDs) have become an issue of worldwide concern and have been clearly identified as a problem to be addressed according to the Sustainable Development Goals by the United Nations [1]. Additionally, NCDs are predicted to cause half of all deaths in most developing countries by 2030 [2]. These countries are also disproportionately affected by communicable diseases [3]; therefore, preventive measures are needed to minimize the double burden of communicable and non-communicable diseases.

Since anti-retroviral therapy (ART) was introduced to treat human immunodeficiency virus (HIV) infection, comorbidities affecting individuals with HIV infection have changed dramatically, with increasing prevalence of overweight and obesity [4] and NCDs [5]. HIV-infected individuals are no longer seriously affected by wasting syndrome; instead, the prevalence of overweight/obesity has been increasing [4, 6]. Such changes necessitate measures to prevent NCDs that target individuals with HIV infection as well as the general population, particularly in sub-Saharan Africa where more than two thirds of the global population of HIV-infected individuals resides [7]. Previous studies in Kenya have reported a lower prevalence of overweight/obesity and hypertension among HIV-infected people than in the general population and in those without HIV infection [8, 9]. However, the situation among Kenyans with HIV infection may have changed since the publication of those reports, as has been the case in South Africa [10, 11]. Overweight/obesity is a known risk factor for NCDs and is an important issue to address in NCD prevention. Yet challenges remain with respect to reducing overweight/obesity among HIV-infected individuals in African countries, where a plump figure is desirable [12, 13] and where AIDS has been thought of as a wasting syndrome [14].

In this study, we aimed to determine the prevalence of overweight/obesity and abdominal obesity as well as hypertension among HIV-infected individuals in Western Kenya and to identify the risk factors of overweight/obesity, abdominal obesity, and hypertension.

## Methods

### Study design

We conducted a cross-sectional study in Mbita, Homa Bay County, Kenya, from September to December 2015. HIV-infected adults were recruited at Mbita Sub-county Hospital and were administered a structured questionnaire as well as anthropometric (weight, height, and waist circumference) and blood pressure measurement.

### Participants and sample size

HIV-infected adults in this study were defined as individuals aged 18 years and older, who had tested positive for HIV infection, were enrolled at the Patient Support Center (PSC) in Mbita Sub-county Hospital for more than 1 year at the time of the study, and had medical records available that included the results of HIV viral load counts within the previous 18 months. All eligible participants at the PSC on the research day were recruited. We excluded individuals who were pregnant; taking hormonal medication, except for contraceptives; and those who were hospitalized.

The sample size was calculated based on the expected prevalence obtained from a previous study conducted among HIV-infected people in Western Kenya where the overall prevalence of overweight and obesity was 18.3% in both men and women [9]. We applied the formula of sample size for a prevalence study with a cross-section design [15], with 0.183 as the expected prevalence, 95% confidence interval (CI) of 1.96, and a margin of error of 0.05 for precision. The calculated sample size was 227. Considering a 10% non-response rate, 251 participants were recruited during the study period.

### Variables

The main dependent variables were body mass index (BMI), waist circumference (WC), and blood pressure. Weight was measured to the nearest 0.1 kg, height to the nearest 1 mm, and WC to the nearest 1 cm. Blood pressure was measured once using a digital sphygmomanometer (HEM-7132; OMRON, Kyoto, Japan). BMI ≤ 18.5 was classified as underweight, BMI ≥ 25 as overweight, and BMI ≥ 30 as obese [16]. For the analysis, overweight and obesity were combined for outcome and designated as overweight. Abdominal obesity was defined as WC ≥ 94 cm for men and ≥ 80 cm for women [17]. The definition of hypertension followed the World Health Organization (WHO) criteria, with diastolic pressure ≥ 90 mmHg or systolic pressure ≥ 140 mmHg [18].

Independent variables were collected using a questionnaire and medical records. The questionnaire comprised demographic information including socioeconomic status (SES), dietary habits, physical activity, and perception of body size in relation to HIV/AIDS. Alcohol use and smoking were also queried (yes or no) in the questionnaire. The following clinical variables were obtained from the medical records: WHO clinical stage of HIV/AIDS [19], intake and duration of ART and protease inhibitors (PIs), CD4 counts, viral load, history of opportunistic infections, and history of the use of tuberculosis medication and contraceptives. We collected information of any type of opportunistic infection noted by a clinician in participants’ medical records.

In the analysis, education level was recategorized as follows: “No education” and “Incomplete primary school” were recategorized into “low education,” “Complete primary school” was retained, and “Complete secondary school” and “higher education” were recategorized into “Complete secondary or higher.” Occupation was also recategorized according to level of the physical activity as follows. “Not working,” “Housework,” and “Student” were recategorized into “At home”; “Farmer” and “Fisherman” into “Working outside”; and “Office work,” “Engineer,” “Small business entrepreneur,” and “Tailor” into “Working inside.” Other occupations including driver and carpenter were retained as “others.” An SES indicator was generated by summing asset ownership, after principal component analysis was applied to weight each asset (car, refrigerator, television, iron, mobile phone, radio, bicycle, sofa, livestock, and poultry). This was divided into four categories according to 25th, 50th, and 75th percentiles and labeled in order from “poorest,” “poor,” “less poor,” to “rich.”

The questionnaire was translated into the local language, Dholuo, then back-translated into English to check for consistency. The questionnaire was modified as required after pre-testing among 40 people who were eligible for participation in the study.

### Physical activity level

Physical activity level during the previous 7 days was assessed using the self-administered International Physical Activity Questionnaire for young and middle-aged adults [20]. This instrument has been validated in many countries, including South Africa [20, 21]. Physical activity was categorized into three levels: inactive, minimally active, and health-enhancing physical activity (HEPA).

### Perception of body size (body image)

Participants’ perception of body size in relation to HIV/AIDS was assessed using nine illustrations of different body figures, which was presented by Lynch et al. in 2009 [22].

### Statistical analysis

Descriptive analysis was conducted to determine the prevalence of overweight, abdominal obesity, and hypertension along with stratification by age and sex, and for key characteristics among participants. First, the association of overweight, abdominal obesity, and hypertension with each independent variable was examined using bivariate analysis by calculating the crude odds ratio (OR) and 95% CI. Then, the OR of each variable was adjusted by age, sex, and SES in multivariable logistic regression analysis to identify the adjusted odds ratio (aOR) and 95% CI. The statistical significance was set as *P* value = 0.05. The statistical analysis was conducted using MedCalc version 19.1.7.

## Results

### Background data of participants

A total 251 participants were enrolled from among those registered at PSC in Mbita Sub-county Hospital. As the total number of those registered at this hospital as of September to December in 2015 was unavailable, it was difficult to determine the response rate. Participants’ characteristics are shown in Table 1. The median age was 38 years with an interquartile range of 32–45 years. All participants had taken ART, most for 1 year or longer. Approximately 75% of participants had a history of tuberculosis, and fewer than 20% had a past history of other opportunistic infections. Viral load was less than 150 copies/ml in approximately 80% of participants.

**Table 1:** Participant characteristics

		Number	Percentage			Number	Percentage
Age	18–19	1	0.4	Socioeconomic status	Poorest	63	25.1
	20–29	32	12.7		Poor	62	24.7
	30–39	104	41.4		Less poor	62	24.7
	40 and older	114	45.4		Rich	63	25.1
					Missing data	1	
Sex	Male	82	32.7	Alcohol	No	223	86.7
	Female	169	67.3				
Education level	No education	11	4.4		Yes	24	9.7
					Missing data	4	
	Low education	85	33.9	Smoke	No	242	96.4
	Complete primary	117	46.6				
	Complete secondary	30	12.0		Yes	9	3.6
	Higher education	8	3.2	Contraceptive use	No	71	28.3
Occupation	Not working	26	10.4				
					Yes	180	71.7
	Housework	5	2.0		Missing data	4	
	Farmer	17	6.8	Opportunistic infection history	No	204	81.9
	Fisherman	31	12.4				
	Office work	3	1.2		Yes	45	18.1
	Engineer	1	0.4		Missing data	2	
	Small entrepreneur	125	49.8	Tuberculosis history	No	66	26.3
	Tailor	7	2.8		Yes	185	73.7
	Student	0	0.0	CD4 counts (cells/μl)	Less than 200	30	12.1
	Other	36	14.3				
On anti-retroviral therapy (ART)	No	0	0.0				
	Yes	251	100.0		200–499	113	45.4
					500 and higher	106	42.6
ART duration	0–14 days	0	0.0		Missing data	2	
	14–55 days	1	0.4	Viral load (copies/ml)	Less than 150	206	82.4
	56–181 days	0	0.0				
	182–364 days	13	5.2		150 and higher	44	17.6
	365 days and more	237	94.4		Missing data	1	
On protease inhibitors	No	214	85.3	WHO stage	1	51	20.3
	Yes	36	14.3		2	71	28.3
	Missing data	1			3	97	38.6
Protease inhibitor duration	0–539 days	7	2.8		4	29	11.6
					Missing data	3	
	540 days and more	28	11.2				
	Missing data	1					

*N* number of study participants, *n* number of elements in a sample. Alcohol use and smoking were also asked as yes or no in the questionnaire. Contraceptive use: hormonal contraceptive use among women. WHO stage: WHO clinical staging of HIV/AIDS for adults and adolescents

### Prevalence of overweight, abdominal obesity, and hypertension

More HIV-infected women were overweight than underweight; only 8.3% of HIV-infected women were underweight whereas 17.2% were overweight and 3.6% were obese. Among HIV-infected men, slightly more were underweight (12.2%) than overweight (11.0%), and none were obese (data not shown in the table).

The prevalence of overweight, abdominal obesity, and hypertension according to different sex and age groups is shown in Table 2. The prevalence of overweight was higher at age 30 years and older. Abdominal obesity was much more common in women (62.1%) than in men (9.6%), with nearly 70% of women aged 30–39 years having abdominal obesity. The prevalence of hypertension in all age groups was 9.8% and 11.8% among men and women, respectively. Men tended to develop hypertension at early ages.

**Table 2:** Prevalence of overweight, abdominal obesity, and hypertension according to sex and age group

	Overweight			Abdominal obesity			Hypertension		
Age	*N*	*n*	%	*N*	*n*	%	*N*	*n*	%
Male									
All ages	82	9	11.0	82	7	8.5	82	8	9.8
18–19	0	0	0	0	0	0.0	0	0	0.0
20–29	7	0	0	7	1	14.3	7	1	14.3
30–39	28	3	10.7	34	2	5.9	28	4	14.3
40 and older	47	6	12.8	41	5	12.2	47	3	6.4
Female									
All ages	169	35	20.7	169	105	62.1	169	20	11.8
18–19	1	0	0	1	0	0.0	1	0	0
20–29	25	3	12.0	25	15	60	25	2	8.0
30–39	76	17	22.4	70	48	68.6	76	4	5.3
40 and older	67	15	22.4	73	42	57.5	67	14	20.9

Overweight includes obesity. Hypertension includes both high systolic blood pressure and diastolic blood pressure. *N* number of study participants, *n* number of elements in a sample

### Factors associated with overweight, abdominal obesity, and hypertension

The results of bivariate and multivariate analysis for overweight are shown in Table 3. A history of opportunistic infections was significantly associated with overweight (OR 2.46, 95% CI 1.10–5.50, *P* = 0.028). Overweight was more common in women (19.5%) than in men (11.0%), although no association was identified.

**Table 3:** Factors associated with overweight

	*N*	*n*	%	OR	95% CI		*P* value	aOR	95% CI		*P* value
Age category											
18–29	33	4	12.1	1.00				1.00			
30–39	104	20	19.2	1.72	0.54	5.47	0.353	1.99	0.61	6.46	0.251
40 and older	114	18	15.8	1.35	0.42	4.33	0.604	1.43	0.44	4.66	0.545
Sex											
Male	82	9	11.0	1.00				1.00			
Female	169	33	19.5	1.96	0.89	4.33	0.093	2.11	0.94	4.72	0.070
Marital status											
Single	7	2	28.6	1.00				1.00			
Married	154	25	16.2	0.48	0.08	2.63	0.402	0.53	0.09	3.05	0.478
Divorced	10	1	10.0	0.27	0.01	3.88	0.341	0.38	0.02	5.69	0.484
Widowed	80	14	17.5	0.53	0.09	3.02	0.474	0.58	0.09	3.51	0.558
Education level											
Low education	96	14	14.6	1.00				1.00			
Complete primary	117	22	18.8	1.35	0.65	2.82	0.414	1.59	0.74	3.44	0.229
Complete secondary or higher	38	6	15.8	1.09	0.38	3.11	0.859	1.59	0.50	5.04	0.429
Occupation											
At home	31	5	16.1	1.00				1.00			
Working outside	48	10	20.8	1.36	0.41	4.47	0.603	1.36	0.40	4.59	0.620
Working inside	136	20	14.7	0.89	0.31	2.61	0.841	0.83	0.27	2.47	0.738
Others	36	7	19.4	1.26	0.35	4.44	0.724	1.29	0.34	4.82	0.695
SES											
Poorest	63	13	20.6	1.00				1.00			
Poor	62	9	14.5	0.65	0.25	1.66	0.371	0.67	0.26	1.74	0.419
Less poor	62	13	21.0	1.02	0.43	2.42	0.963	0.95	0.39	2.31	0.926
Rich	63	7	11.1	0.48	0.17	1.30	0.1491	0.42	0.15	1.16	0.096
Physical activity level											
Inactive	11	2	18.2	1.00				1.00			
Minimally active	22	4	18.2	1.00	0.15	6.53	1	0.92	0.13	6.29	0.933
HEPA active	215	36	16.7	0.91	0.18	4.36	0.9011	0.80	0.15	4.04	0.791
Chai per day											
0–1 cup	20	3	15.0	1.00				1.00			
2 cups	58	11	19.0	1.32	0.32	5.33	0.691	1.19	0.28	4.94	0.803
3 cups or more	173	28	16.2	1.09	0.30	3.98	0.8913	0.96	0.25	3.63	0.963
Soda per week											
None	126	23	18.3	1.00				1.00			
Once	64	7	10.9	0.55	0.22	1.36	0.196	0.49	0.19	1.25	0.141
Twice or more	61	12	19.7	1.10	0.50	2.38	0.8157	0.99	0.44	2.21	0.984
Ugali per day											
Quarter a plate	185	32	17.3	1.00				1.00			
Half a plate	55	8	14.5	0.81	0.35	1.88	0.631	0.97	0.40	2.31	0.946
3 quarters a plate or more	8	1	12.5	0.68	0.08	5.74	0.7257	0.74	0.08	6.60	0.789
Alcohol											
No	223	35	15.7	1.00				1.00			
Yes	24	5	20.8	1.41	0.49	4.03	0.5179	1.66	0.54	5.04	0.367
Smoke											
No	242	41	16.9	1.00				1.00			
Yes	9	1	11.1	0.61	0.07	5.03	0.648	0.91	0.10	8.20	0.935
Abdominal obesity											
No	138	21	15.2	1.00				1.00			
Yes	113	21	18.6	1.27	0.65	2.46	0.477	0.94	0.43	2.03	0.885
Hypertension											
No	222	35	15.8	1.00				1.00			
Yes	28	7	25.0	1.78	0.70	4.51	0.223	2.04	0.77	5.39	0.147
ART duration											
0–364 days	14	4	28.6	1.00				1.00			
365 days and more	237	38	16.0	0.48	0.14	1.60	0.231	0.52	0.15	1.84	0.314
CD4 counts (cells/μl)											
< 200	30	3	10.0	1.00				1.00			
200–499	113	20	17.7	1.93	0.53	7.01	0.315	2.08	0.55	7.79	0.276
≥ 500	106	19	17.9	1.96	0.54	7.15	0.3053	1.94	0.51	7.31	0.326
On PI											
No	214	39	18.2	1.00				1.00			
Yes	36	2	5.6	0.41	0.11	1.39	0.1537	0.45	0.12	1.57	0.323
PI duration											
0–359 days	7	1	14.3	1.00				1.00			
540 days and more	28	2	7.1	0.46	0.03	5.96	0.553				0.999
WHO stage											
1	51	8	15.7	1.00				1.00			
2	71	17	23.9	1.69	0.66	4.29	0.268	1.85	0.71	4.86	0.208
3	97	12	12.4	0.75	0.28	1.99	0.576	0.74	0.27	2.02	0.563
4	29	5	17.2	1.11	0.32	3.81	0.8562	1.04	0.28	3.79	0.946
OI history											
No	204	30	14.7	1.00				1.00			
Yes	45	12	26.7	2.11	0.98	4.53	0.0562	2.46	1.10	5.50	0.028

*N* number of study participants, *n* number of elements in a sample, *OR* odds ratio, *95% CI* 95% confidential interval, *aOR* adjusted odds ratio, *P value* probability value, *HEPA* health-enhancing physical activity, *ART* anti-retroviral therapy, *PI* protease inhibitor, *WHO stage* WHO clinical staging of HIV/AIDS for adults and adolescent, *OI* opportunistic infection

The results of bivariate and multivariate analysis for abdominal obesity are shown in Table 4. We identified an association between abdominal obesity and female sex (aOR 15.28, 95% CI 6.84–34.12, *P* < 0.0001). Abdominal obesity was more common in participants with a history of opportunistic infections (53.3%) than in those without this history (43.1%), although no association was identified. Other factors including level of physical activity was not significantly associated with either overweight or abdominal obesity.

**Table 4:** Factors associated with abdominal obesity

	*N*	*n*	%	OR	95% CI		*P* value	aOR	95% CI		*P* value
Age category											
18–29	33	16	48.5	1.00				1.00			
30–39	104	50	48.1	0.98	0.44	2.15	0.967	1.30	0.53	3.19	0.558
40 and older	114	47	41.2	0.74	0.34	1.66	0.459	1.06	0.44	22.54	0.896
Sex											
Male	82	8	9.8	1.00				1.00			
Female	169	105	62.1	15.17	6.86	33.53	< 0.0001	15.28	6.84	34.12	< 0.0001
Marital status											
Single	7	3	42.9	1				1			
Married	154	71	46.1	1.14	0.24	5.26	0.866	1.94	0.35	10.57	0.441
Divorced	10	4	40.0	0.88	0.12	6.31	0.906	2.11	0.21	20.42	0.516
Widowed	80	35	43.8	1.03	0.21	4.93	0.963	2.18	0.37	12.68	0.382
Education level											
Low education	96	41	42.7	1.00				1.00			
Complete primary	117	51	43.6	1.03	0.60	1.78	0.897	0.86	0.45	1.67	0.676
Complete secondary or higher	38	21	55.3	1.65	0.77	3.53	0.19	1.34	0.50	3.61	0.551
Occupation											
At home	31	15	48.4	1.00				1.00			
Working outside	48	23	47.9	0.98	0.39	2.42	0.967	0.79	0.26	2.40	0.685
Working inside	136	57	41.9	0.76	0.35	1.68	0.511	0.63	0.24	1.67	0.360
Others	36	18	50.0	1.06	0.40	2.78	0.895	0.57	0.17	1.85	0.357
SES											
Poorest	63	23	36.5	1.00				1.00			
Poor	62	23	37.1	1.02	0.49	2.12	0.945	0.99	0.43	2.28	0.990
Less poor	62	32	51.6	1.85	0.90	3.79	0.090	1.54	0.68	3.51	0.294
Rich	63	35	55.6	2.17	1.06	4.44	0.033	1.99	0.86	4.60	0.104
Physical activity level											
Inactive	11	6	54.5	1.00							
Minimally active	22	9	40.9	0.57	0.13	2.48	0.46	0.40	0.07	2.28	0.302
HEPA active	215	97	45.1	0.68	0.20	2.31	0.542	0.55	0.12	2.40	0.429
Chai per day											
0–1 cup	20	8	40.0	1.00				1.00			
2 cups	58	27	46.6	1.30	0.46	3.66	0.611	1.14	0.32	4.05	0.836
3 cups or more	173	78	45.1	1.23	0.47	3.16	0.665	0.87	0.27	2.79	0.827
Soda per week											
None	126	58	46.0	1.00				1.00			
Once	64	27	42.2	0.85	0.46	1.57	0.614	0.66	0.32	1.34	0.255
Twice or more	61	28	45.9	0.99	0.53	1.83	0.986	0.96	0.46	2.03	0.933
Ugali per day											
Quarter a plate	185	91	49.2	1.00				1.00			
Half a plate	55	19	34.5	0.54	0.29	1.01	0.057	0.60	0.28	1.27	0.186
3 quarters a plate or more	8	2	25.0	0.34	0.06	1.75	0.198	0.36	0.04	1.27	0.205
Alcohol											
No	223	103	46.2	1.00				1.00			
Yes	24	8	33.3	0.58	0.23	1.41	0.233	1.16	0.38	3.52	0.792
Smoke											
No	242	112	46.3	1.00				1.00			
Yes	9	1	11.1	0.14	0.01	1.17	0.07	0.52	0.04	5.54	0.590
Abdominal obesity											
No	209	92	44.0	1.00				1.00			
Yes	42	21	50.0	1.27	0.65	2.46	0.477	0.95	0.44	2.09	0.900
Hypertension											
No	222	97	43.7	1.00				1.00			
Yes	28	16	57.1	1.71	0.77	3.80	0.181	1.72	0.65	4.53	0.272
ART duration											
0–364 days	14	9	64.3	1.00				1.00			
365 days and more	237	104	43.9	0.43	0.14	1.33	0.145	0.34	0.08	1.39	0.136
CD4 counts (cells/μl)											
< 200	30	15	50.0	1.00				1.00			
200–499	113	50	44.2	0.79	0.35	1.77	0.574	1.04	0.41	2.65	0.927
≥ 500	106	48	45.3	0.82	0.36	1.86	0.647	1.07	0.41	2.75	0.885
On PI											
No	214	95	44.4	1.00				1.00			
Yes	36	18	50.0	1.25	0.61	2.53	0.532	1.82	0.74	4.44	0.187
PI duration											
0–359 days	7	3	42.9	1.00				1.00			
540 days and more	28	15	53.6	1.53	0.28	8.18	0.613	0.04	0.00	2.60	0.131
WHO stage											
1	51	20	39.2	1.00				1.00			
2	71	37	52.1	1.68	0.81	3.50	0.160	2.37	0.97	5.73	0.056
3	97	40	41.2	1.08	0.54	2.17	0.811	1.14	0.51	2.58	0.736
4	29	15	51.7	1.66	0.66	4.16	0.279	1.85	0.61	5.57	0.269
OI history											
No	204	88	43.1	1.00				1.000			
Yes	45	24	53.3	1.50	0.78	2.87	0.215	2.21	0.97	5.02	0.057

*N* number of study participants, *n* number of elements in a sample, *OR* odds ratio, *95% CI* 95% confidential interval, *aOR* adjusted odds ratio, *P value* probability value, *HEPA* health-enhancing physical activity, *ART* anti-retroviral therapy, *PI* protease inhibitor, *WHO stage* WHO clinical staging of HIV/AIDS for adults and adolescents, *OI* opportunistic infection

The results of bivariate and multivariate analysis with hypertension are shown in Table 5. We identified an association between hypertension and WHO clinical stage. WHO clinical stage 3 was less strongly associated with hypertension than WHO clinical stage 1 (aOR 0.18, 95% CI 0.05–0.58, *P* < 0.01), as was the case for WHO clinical stage 4 in comparison with WHO clinical stage 1 (aOR 0.16, 95% CI 0.02–0.87, *P* < 0.05).

**Table 5:** Factors associated with hypertension

	*N*	*n*	%	OR	95% CI		*P* value	aOR	95% CI		*P* value
Age category											
18–29	33	3	9.1	1.00				1.00			
30–39	104	8	7.7	0.83	0.21	3.34	0.797	0.75	0.18	3.08	0.308
40 and older	113	17	15.0	1.77	0.48	6.45	0.387	1.93	0.51	7.23	0.952
Sex											
Male	81	8	9.9	1.00				1.00			
Female	169	20	11.8	1.22	0.51	2.91	0.646	1.23	0.51	2.99	0.645
Marital status											
Single	7	0	0.0	1.00				1.00			
Married	154	17	11.0				0.999				0.999
Divorced	10	0	0.0	1.00			1.000				1.000
Widowed	79	11	13.9	1.00			0.999				0.999
Education level											
Low education	96	13	13.5	1.00				1.00			
Complete primary	116	11	9.5	0.66	0.28	1.56	0.355	0.59	0.23	1.47	0.260
Complete secondary or higher	38	4	10.5	0.75	0.22	2.46	0.637	0.42	0.11	1.71	0.229
Occupation											
At home	31	2	6.5	1.00				1.00			
Working outside	48	4	8.3	1.31	0.22	7.66	0.759	1.35	0.22	8.11	0.741
Working inside	135	17	12.6	2.08	0.45	9.55	0.342	1.98	0.42	9.35	0.387
Others	36	5	13.9	2.33	0.42	13.01	0.332	1.79	0.31	10.49	0.516
SES											
Poorest	63	7	11.1	1.00				1.00			
Poor	62	4	6.5	0.55	0.15	1.98	0.363	0.51	0.14	1.86	0.308
Less poor	62	7	11.3	1.01	0.33	3.09	0.975	0.96	0.31	2.98	0.952
Rich	62	10	16.1	1.53	0.54	4.33	0.414	1.78	0.61	5.17	0.289
Physical activity level											
Inactive	11	2	18.2	1.00				1.00			
Minimally active	22	2	9.1	0.45	0.05	3.71	0.459	0.46	0.05	4.01	0.482
HEPA active	214	24	11.2	0.56	0.11	2.78	0.486	0.46	0.08	2.43	0.364
Chai per day											
0–1 cup	20	0	0.0	1.00				1.00			
2 cups	58	1	1.7				0.998				0.998
3 cups or more	172	27	15.7				0.997				0.998
Soda per week											
None	125	19	15.2	1.00				1.00			
Once	64	4	6.3	0.37	0.12	1.14	0.085	0.41	0.13	1.29	0.130
Twice or more	61	5	8.2	0.49	0.17	1.41	0.188	0.57	0.20	1.66	0.311
Ugali per day											
Quarter a plate	184	24	13.0	1.00				1.00			
Half a plate	55	4	7.3	0.52	0.17	1.57	0.250	0.50	0.16	1.54	0.230
3 quarters a plate or more	8	0	0.0				0.998				0.998
Alcohol											
No	222	24	10.8	1.00				1.00			
Yes	24	3	12.5	1.17	0.32	4.24	0.802	1.35	0.35	5.23	0.661
Smoke											
No	241	28	11.6	1.00				1.00			
Yes	9	0	0.0				0.998				0.998
Overweight/obesity											
No	208	21	10.1	1.00				1.00			
Yes	42	7	16.7	1.78	0.70	4.51	0.223	1.95	0.74	5.13	0.173
Abdominal obesity											
No	137	12	8.8					1.00			
Yes	113	16	14.2					1.71	0.66	4.42	0.261
ART duration											
0–364 days	14	2	14.3	1.00				1.00			
365 days and more	236	26	11.0	1.71	0.77	3.80	0.182	0.51	0.10	2.59	0.424
CD4 counts (cells/μl)											
< 200	30	3	10.0	1.00				1.00			
200–499	113	13	11.5	1.17	0.31	4.40	0.816	1.51	0.38	5.95	0.555
≥ 500	105	12	11.4	1.16	0.31	4.41	0.826	1.62	0.40	6.56	0.495
On PI											
No	214	26	12.1	1.00				1.00			
Yes	35	2	5.7	0.43	0.09	1.93	0.276	0.39	0.08	1.81	0.234
PI duration											
0–359 days	7	0	0.0	1.00				1.00			
540 days and more	27	2	7.4				0.998				0.999
WHO stage											
1	51	10	19.6	1.00				1.00			
2	71	10	14.1	0.67	0.25	1.75	0.414	0.52	0.18	1.44	0.211
3	96	6	6.3	0.27	0.09	0.80	0.018	0.18	0.05	0.58	0.004
4	29	2	6.9	0.30	0.06	1.49	0.143	0.16	0.02	0.87	0.034
OI history											
No	204	21	10.3	1.00				1.00			
Yes	44	7	15.9	1.64	0.65	4.15	0.290	1.52	0.57	4.00	0.396

*N* number of study participants, *n* number of elements in a sample, *OR* odds ratio, *95% CI* 95% confidential interval, *aOR* adjusted odds ratio, *P value* probability value, *HEPA* health-enhancing physical activity, *ART* anti-retroviral therapy, *PI* protease inhibitor, *WHO stage* WHO clinical staging of HIV/AIDS for adults and adolescents, *OI* opportunistic infection

### Perceptions of body size

More than half of HIV-infected participants (*n* = 150; 60%) assumed that the thinnest among the nine body figures was indicative of HIV infection whereas 37.6% (*n* = 94) did not make this assumption for any of the body figures. Only 1.6 % (*n* = 4) assumed that the largest figure was indicative of HIV infection. Perception of a thin body figure as indicating HIV infection was significantly associated with personal experience of or witnessing discrimination against HIV-infected people (OR 3.67, 95% CI 2.36–5.69, *P* < 0.001). However, no association was found between an experience of discrimination and overweight (OR 1.84, 95% CI 0.94–3.16, *P* = 0.070) or experience of discrimination and abdominal obesity (OR 1.25, 95% CI 0.74–2.12, *P* = 0.398).

## Discussion

The findings of this study highlight the current situation regarding body weight and blood pressure among HIV-infected individuals in Western Kenya.

Overweight was much more prevalent than underweight among women in this study. Moreover, we observed a high prevalence of abdominal obesity among women. First, the second regimen of ART, or PIs, is known to cause metabolic side effects including abdominal obesity [23]; however, only a few individuals in our study population had taken PIs. A high prevalence of abdominal obesity among HIV-infected women who were not taking PIs is compatible with the findings of a previous study reporting a positive association between abdominal obesity and HIV infection with an ART regimen, even with minor metabolic side effects [24]. Second, abdominal obesity among women was defined as WC ≥ 80 cm [17]; however, this cutoff point is controversial [25–29]. The WC cutoff point for abdominal obesity among sub-Saharan African populations is based on the cutoff point obtained from studies conducted among European populations [17]. A study targeting HIV-infected people in South Africa determined that 90 cm was an optimal cutoff point [29]. Furthermore, the combined use of WC and hip circumference could more effectively predict an increased health risk among HIV-infected individuals than use of WC alone [30]. Therefore, further investigation using different cutoff points and measurement methods is desirable.

Only female sex was found to be a risk factor of abdominal obesity, which is in agreement with a previous study [24]. In our study, no other potential associations were found for other dependent variables such as high SES, being married, and older age, as have been reported in previous studies [10, 31]. A possible reason for this might be bias and a small study population with similar lifestyles and economic levels. Consumption of sugar-sweetened beverages is a well-known risk factor of overweight/obesity in the general population [32]; however, we found no association between consumption of soda or chai and overweigh/obesity in our study population, despite the fact that drinking chai is an integral aspect of Kenyan culture. Physical activity was not found to be associated with a decreased risk of overweight/obesity in this study, despite being widely known as a preventive factor for overweight/obesity and hypertension [33]. Most participants had HEPA levels of physical activity, which implies that people throughout the study area have similar lifestyles. This could explain the lack of an association between chai or soda consumption and overweight/obesity, as high energy consumption was accompanied by high activity levels.

An association of overweight with a history of opportunistic infections was revealed in this study. Studies on the association between overweight/obesity and opportunistic infections are limited, and the precise relationship between them remains unknown. However, a previous report noted that the presence of an opportunistic infection decreased the likelihood of overweight/obesity [34]. Furthermore, an association between higher BMI and higher CD4 counts was reported in a past study [35], which may lessen the likelihood of acquiring an opportunistic infection. However, some evidence of a relationship between obesity and inflammation, such as surgical-site infections, nosocomial infections, periodontitis, and skin infections, has been established [36]. In our study, a causal relationship could not be established owing to the cross-sectional study design. There is a possibility that participants had opportunistic infections at the time of HIV diagnosis or on beginning ART. Then, opportunistic infections were treated and the individual regained weight afterward because ART initiation has been reportedly associated with overweight/obesity [4]. Further research is needed to follow weight change and the occurrence of opportunistic infections or other events, to identify the risk and outcomes of overweight/obesity.

In our study population, perceptions about body size may remain problematic. Most people associated the thinnest body figure as indicative of HIV infection, having remembered wasting disease during the initial stages of the HIV/AIDS outbreak [14]. This perception might also be reinforced by experiences of discrimination or observing discrimination against people who are thin. In fact, participants who had such experiences were more likely to perceive a thin body figure as indicative of HIV infection.

The findings of this study highlight the necessity of intervention to prevent and decrease overweight and abdominal obesity among people living with HIV as well as among the general population. Our study adds several important points to the knowledge base in this regard. Understanding local beliefs and concepts is important when introducing any new approach to health. Continuous monitoring and further investigation are also necessary because the physical condition of HIV-positive individuals, including their nutritional status, may change according to ART outcome and lifestyle changes.

This study has several limitations. First, we did not include an HIV-negative group for comparison, so it was difficult to identify those factors only affecting people with HIV infection. Second, the data used in this study do not represent the population of the entire study area because the study location and participants were not randomly selected. Third, we failed to recruit a sufficient number of participants to conduct efficient statistical analysis because of the limited study period. Lastly, owing to technical differences and time management issues, we could not measure hip circumference, to obtain the waist-to-hip ratio, which is often used to assess cardiovascular risk in people with HIV infection. Despite these limitations, our study provides informative insights, adding to the knowledge gained in early studies on NCDs and HIV infection on the African continent.

## Conclusion

HIV-infected women were more likely to be overweight or obese than underweight whereas the men with HIV infection in our study tended to be underweight. Abdominal obesity was much common in women, especially among those in their 30s. Female sex and a history of opportunistic infections were identified as risk factors of abdominal obesity and overweight, respectively, and we identified a negative association between hypertension and WHO clinical stage. More than half of participants assumed a thin body figure indicated HIV infection, although none of the assessed factors was significantly associated with this assumption. HIV-infected individuals, especially women, should be targeted in preventive interventions for overweight and abdominal obesity, with consideration of relevant social and cultural aspects. In addition, continuous monitoring and further investigation are necessary as the physical and clinical condition of people living with HIV, including their nutritional status, may change according to ART outcome and lifestyle changes.

## Data Availability

The datasets used and analyzed during the current study are available from the corresponding author on reasonable request.

## Abbreviations

AIDS: Acquired immune deficiency syndrome
aOR: Adjusted odds ratio
ART: Anti-retroviral therapy
BMI: Body mass index
CI: Confidence interval
HEPA: Health-enhancing physical activity
HIV: Human immunodeficiency virus
NCDs: Non-communicable diseases
OR: Odds ratio
PIs: Protease inhibitors
PSC: Patient Support Center
SES: Socioeconomic status
WC: Waist circumference
WHO: World Health Organization
